# Identifying effective diagnostic biomarkers for childhood cerebral malaria in Africa integrating coexpression analysis with machine learning algorithm

**DOI:** 10.1186/s40001-022-00980-w

**Published:** 2023-02-13

**Authors:** Jia-Xin Li, Wan-Zhe Liao, Ze-Min Huang, Xin Yin, Shi Ouyang, Bing Gu, Xu-Guang Guo

**Affiliations:** 1 Department of Clinical Laboratory Medicine, The Third Affiliated Hospital of Guangzhou Medical University, Guangzhou, 510150 China; 2 Department of Clinical Medicine, The First Clinical School of Guangzhou Medical University, Guangzhou, 511436 China; 3 Department of Clinical Medicine, The Nanshan College of Guangzhou Medical University, Guangzhou, 511436 China; 4 Department of Clinical Medicine, The Third Clinical School of Guangzhou Medical University, Guangzhou, 511436 China; 5 Department of Pediatrics, The Pediatrics School of Guangzhou Medical University, Guangzhou, 511436 China; 6 Department of Infectious Disease, The Fifth Affiliated Hospital of Guangzhou Medical University, Guangzhou, 510150 China; 7 Laboratory Medicine, Guangdong Provincial People’s Hospital (Guangdong Academy of Medical Sciences), Southern Medical University, Guangzhou, 510000 China; 8 Guangdong Provincial Key Laboratory of Major Obstetric Diseases, The Third Affiliated Hospital of Guangzhou Medical University, Guangzhou, 510150 China; 9 Guangzhou Key Laboratory for Clinical Rapid Diagnosis and Early Warning of Infectious Diseases, KingMed School of Laboratory Medicine, Guangzhou Medical University, Guangzhou, China

**Keywords:** Cerebral malaria, WGCNA, Machine learning, Neutrophil, Blood–brain barrier (BBB)

## Abstract

**Background:**

Cerebral malaria (CM) is a manifestation of malaria caused by plasmodium infection. It has a high mortality rate and severe neurological sequelae, existing a significant research gap and requiring further study at the molecular level.

**Methods:**

We downloaded the GSE117613 dataset from the Gene Expression Omnibus (GEO) database to determine the differentially expressed genes (DEGs) between the CM group and the control group. Weighted gene coexpression network analysis (WGCNA) was applied to select the module and hub genes most relevant to CM. The common genes of the key module and DEGs were selected to perform further analysis. The least absolute shrinkage and selection operator (LASSO) logistic regression and support vector machine recursive feature elimination (SVM-RFE) were applied to screen and verify the diagnostic markers of CM. Eventually, the hub genes were validated in the external dataset. Gene set enrichment analysis (GSEA) was applied to investigate the possible roles of the hub genes.

**Results:**

The GO and KEGG results showed that DEGs were enriched in some neutrophil-mediated pathways and associated with some lumen structures. Combining LASSO and the SVM-RFE algorithms, *LEF1* and *IRAK3* were identified as potential hub genes in CM. Through the GSEA enrichment results, we found that *LEF1* and *IRAK3* participated in maintaining the integrity of the blood–brain barrier (BBB), which contributed to improving the prognosis of CM.

**Conclusions:**

This study may help illustrate the pathophysiology of CM at the molecular level. *LEF1* and *IRAK3* can be used as diagnostic biomarkers, providing new insight into the diagnosis and prognosis prediction in pediatric CM.

**Supplementary Information:**

The online version contains supplementary material available at 10.1186/s40001-022-00980-w.

## Introduction

Cerebral malaria (CM), a neurological complication caused by malaria, is one of the most serious neurological disease in the world, with a high mortality rate, and is the primary cause of malaria death [1]. The onset age of CM mainly occurs in children aged 40–45 months, and children under five years of age account for 67% of all malaria deaths [2]. The typical clinical symptoms of CM in children include fever, anorexia, vomiting, cough, convulsions, and coma [3]. It has been reported that approximately 1% of children with Plasmodium falciparum are likely to develop CM. Approximately 11% of childhood survivors of CM have neurological sequelae, such as epilepsy, movement disorders, hemiplegia, speech disorders, cortical blindness, and hypotonia [4]. Therefore, to improve the prognosis of patients, one of the most important factors is early diagnosis [5]. However, due to its complex and nonspecific clinical manifestations, there is no gold standard for the diagnosis of CM [6]. In recent years, the rapid development of bioinformatics technology has greatly promoted research on diagnostic markers of disease. Nowadays, there are many studies on the biomarkers of childhood CM, but there are still few studies on the representative biomarkers of childhood cerebral malaria, and the biomarkers may have population differences [7, 8]. In addition to early diagnosis, effective therapeutic drugs have a critical impact on the prognosis of CM. Recently, the use of quinine or artemisinin derivatives as first-line malaria treatments has significantly reduced Plasmodium infection rates. However, they generally fail to protect against cell death, nerve damage, and cognitive deficits, having less effect on CM [9]. In addition, studies have shown that Plasmodium falciparum resistant to artemisinin derivatives (ART) has emerged in some areas, which is detrimental to the prognosis of patients [10, 11]. To improve the prognosis of patients, it is necessary to understand the molecular mechanisms of the complex biological processes involved in CM.

Therefore, this study applied a variety of bioinformatics tools to study the molecular biological functions, signaling pathway changes and biological targets in the process of CM infection, aiming at discovering the molecular mechanism of the disease and finding key, representative, and highly correlated biomarkers that can be used to diagnose CM and predict the progression and outcome of CM.

## Methods and materials

### Differentially expressed genes (DEGs) screening and functional enrichment analysis

In this study, we downloaded the GSE117613 dataset from the GEO database (https://www.ncbi.nlm.nih.gov/geo/). The GSE117613 dataset included 17 African pediatric CM samples and 12 control samples [12]. These data were normalized using the normalizeBetweenArrays function in the limma package. Subsequently, the limma package was also applied to identify the DEGs between the CM and control groups. Adjusted *p* < 0.05 and log |FC|> 1 were considered the statistical screening criterion. In addition, a volcano map and heatmap were made to display the differential expression of DEGs using the ggplot2 package. Gene Ontology (GO) and Kyoto Encyclopedia of Genes and Genomes (KEGG) enrichment analyses were performed on DEGs to analyze their biological function by the ClusterProfiler R package. GO terms and KEGG pathways with adjusted *p* < 0.05 were deemed statistically significant.

### Weighted gene coexpression network analysis (WGCNA) network construction

WGCNA is a novel method to efficiently detect gene modules and hub genes associated with clinical features [13]. In this study, we performed WGCNA on the GSE117613 gene set using the WGCNA package. The detailed data analysis process is as follows. First, genes in the top 25% of variance were screened for the construction of coexpression networks. Second, hierarchical clustering analysis of the samples was performed to detect and remove outlier samples. Next, according to the WGCNA tutorial, pickSoftThreshold function was applied to determine the best soft-thresholding power to meet the scale-free topology criterion. Thereafter, WGCNA network construction and module detection were performed based on soft-thresholding power and a minimal module size of 200. Eventually, gene significance (GS) and module membership (MM) were calculated to evaluate the correlation between modules and CM. The module with the highest correlation with CM was selected as the key module. The common genes of the key module and DEGs were identified by Venn analysis for further analysis.

### Hub genes identification and validation

To screen diagnostic markers for CM, we applied two machine learning algorithms to identify candidate genes for CM diagnosis. The least absolute shrinkage and selection operator (LASSO) logistic regression is a useful variable selection method by the glmnet R package [14]. LASSO compressed the regression coefficients of some variables to zero by imposing constraints on the model parameters (λ), thereby obtaining an interpretable model. In this process, variables with zero regression coefficients were excluded from the model. Therefore, we applied the LASSO to narrowed down CM-related candidate genes. Furthermore, we utilized support vector machine recursive feature elimination (SVM-RFE) by the e1071 R package [15]. SVM-RFE is a machine learning method for screening genes for sample classification from microarray data. Here, SVM-RFE was performed to identify the value of these biomarkers in CM. The intersection genes of LASSO and SVM-RFE were screened out and then validated in the GSE1124 dataset.

### Gene set enrichment analysis (GSEA)

To further investigate the potential roles of hub genes, we performed GSEA via the gseKEGG function of the clusterProfiler package on hub genes. Based on the median expression levels of hub genes, 17 CM samples in the GSE117613 dataset were divided into low-expression and high-expression groups to perform enrichment analysis. The enrichment pathways with *p*-value < 0.05 and FDR < 25% were considered statistically significant.

## Results

### DEGs filtration and enrichment analysis

**Fig. 1:**
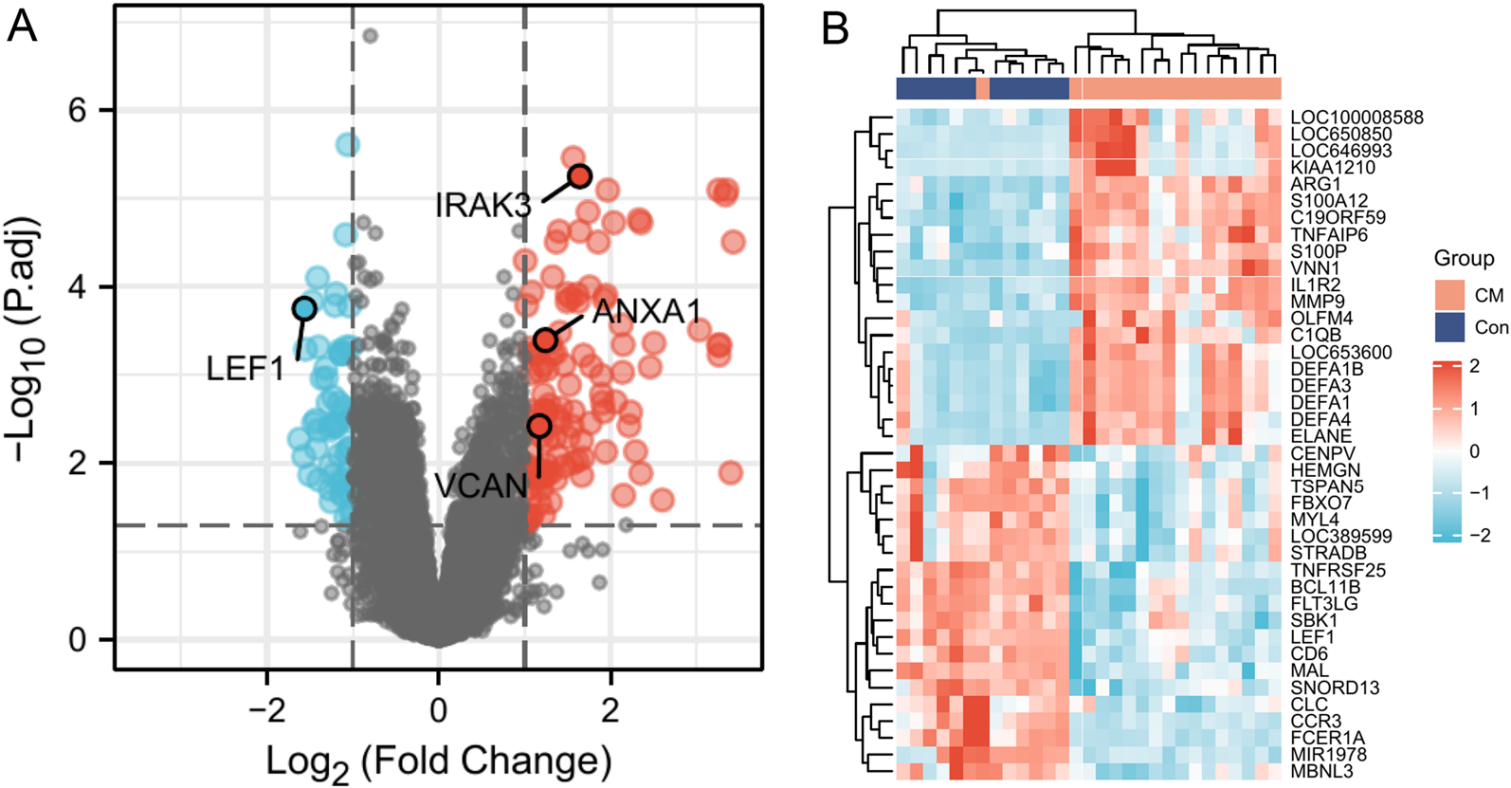
Volcano map and heatmap of differentially expressed genes. **A** DEGs between cerebral malaria blood samples and control samples. Red dot: upregulated gene, blue dot: downregulated gene. **B** The top 20 upregulated genes and top 20 downregulated genes in GSE117613 dataset

**Fig. 2:**
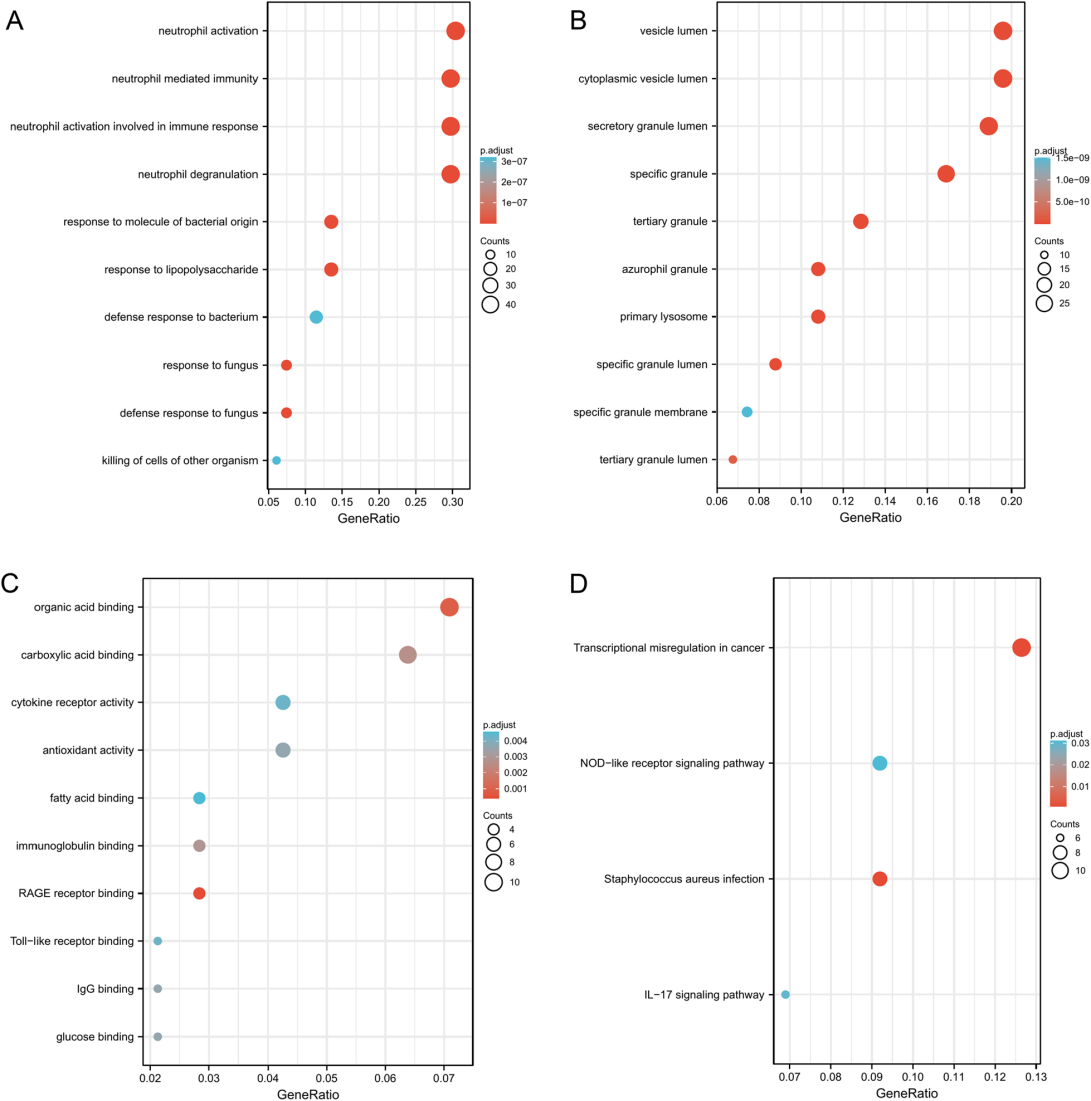
GO and KEGG enrichment analysis. **A** GO enrichment in Biological Process terms. **B** GO enrichment in Cellular Component terms. **C** GO enrichment in Molecular Function. **D** Enriched KEGG pathways of the DEGs

Based on *p*.adj  < 0.05 and log|FC|> 1, 182 DEGs were identified in the GSE117613 dataset, including 124 upregulated and 58 downregulated genes (Fig. 1A). The top 20 upregulated and downregulated DEG profiles are shown in Fig. 1B. GO and KEGG analyses were introduced to analyze the DEGs, thereby identifying their biological functions. GO analysis was composed of biological process (BP), cellular components (CC), and molecular function (MF). For BP, DEGs were enriched in some pathways related to neutrophils, such as neutrophil activation, neutrophil degranulation, neutrophil activation involved in immune response, and neutrophil-mediated immunity (Fig. 2A). For CC, the most significant terms were associated with some lumen structures, such as vesicle lumen and cytoplasmic vesicle lumen (Fig. 2B). For MF, DEGs were enriched in binding-related functions, such as organic acid binding and carboxylic acid binding (Fig. 2C). The significantly enriched KEGG terms were transcriptional misregulation in cancer, the NOD-like receptor signaling pathway, Staphylococcus aureus infection, and the IL-17 signaling pathway (Fig. 2D).

### Weighted gene coexpression network construction and analysis

**Fig. 3:**
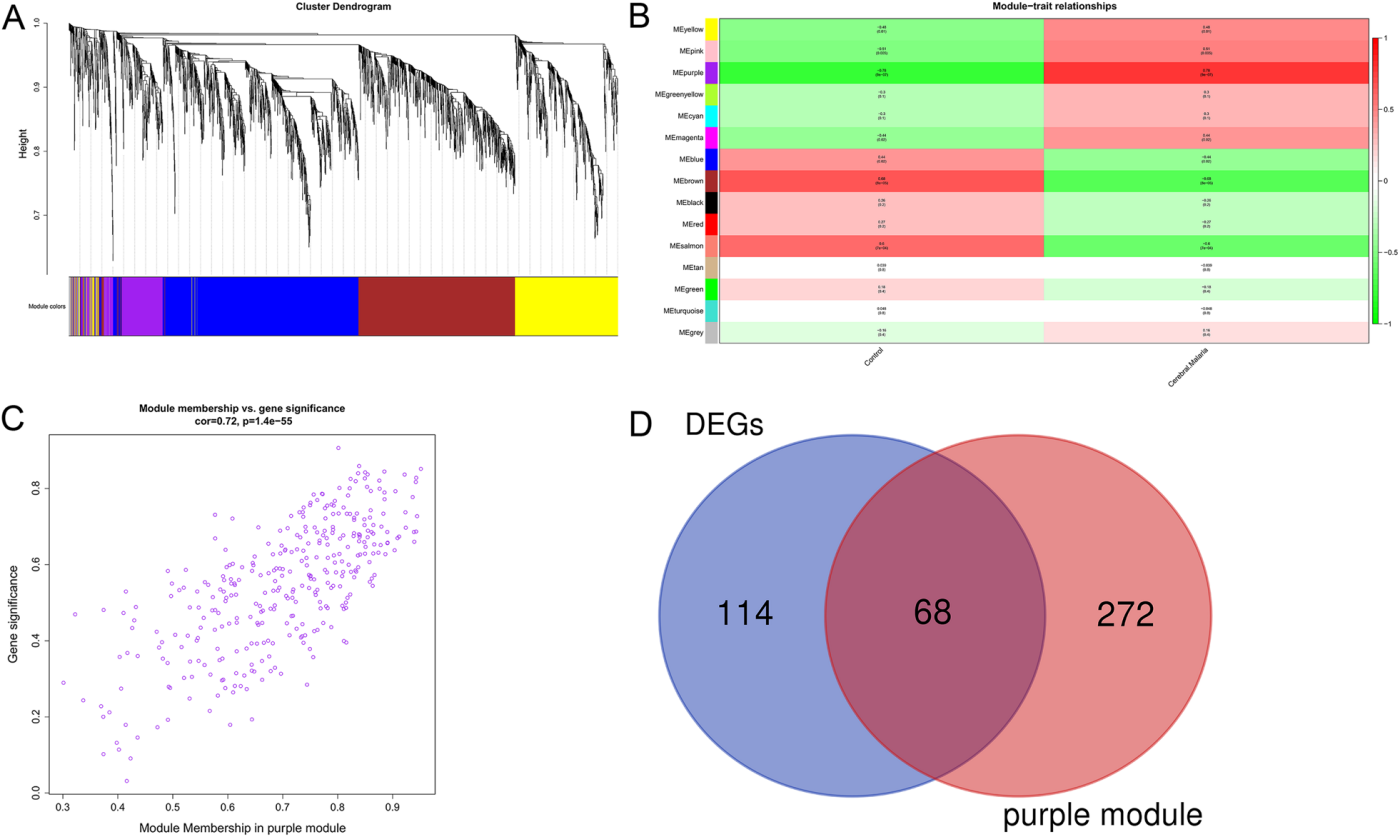
WCGNA coexpression module construction. **A** The cluster dendrogram of the top 25% genes with highest variance in GSE117613. Each specified color represents a specific gene module. The genes in a same module have highly shared biological function. **B** Associations between gene modules and Cerebral Malaria. Each row corresponds to a module eigengene and each column corresponds to a clinical status. Each cell displayed the correlations and *p*-values between each module and clinical status. The purple module was the highest correlation module with cerebral malaria. **C** Scatterplot of gene significance vs. module membership in the purple module. **D** Venn diagram for intersection between DEGs and genes of purple module

Through preliminary screening, a total of 8674 genes, whose variance ranked in the top 25%, were incorporated into the WGCNA network construction, and outlier sample GSM3305179 was excluded after sample cluster analysis. (Additional file 1: Fig. S1). With the pickSoftThreshold algorithm, β = 8 (with R2 = 0.85) was determined as the scale-free topology criterion (Additional file 2: Fig. S2). Then, the coexpression network was established based on soft-thresholding power β = 8 and a cutheight of 0.25. Subsequently, DEGs were clustered into 15 modules (labeled with different colors) with a minimum module size of 200 by hierarchical average linkage clustering (Fig. 3A). The genes in the same modules indicated highly shared biological functions, and unassigned genes were divided into the gray module. The heatmap illustrated the correlation between CM and different modules, in which the purple module was the most highly correlated with CM (cor = 0.78, *p* = 9e−07) (Fig. 3B and C). Therefore, the purple module was considered the key module. The 68 genes shared by the purple module and DEGs were screened out for further analysis (Fig. 3D).

### Hub genes filtration and verification

**Fig. 4:**
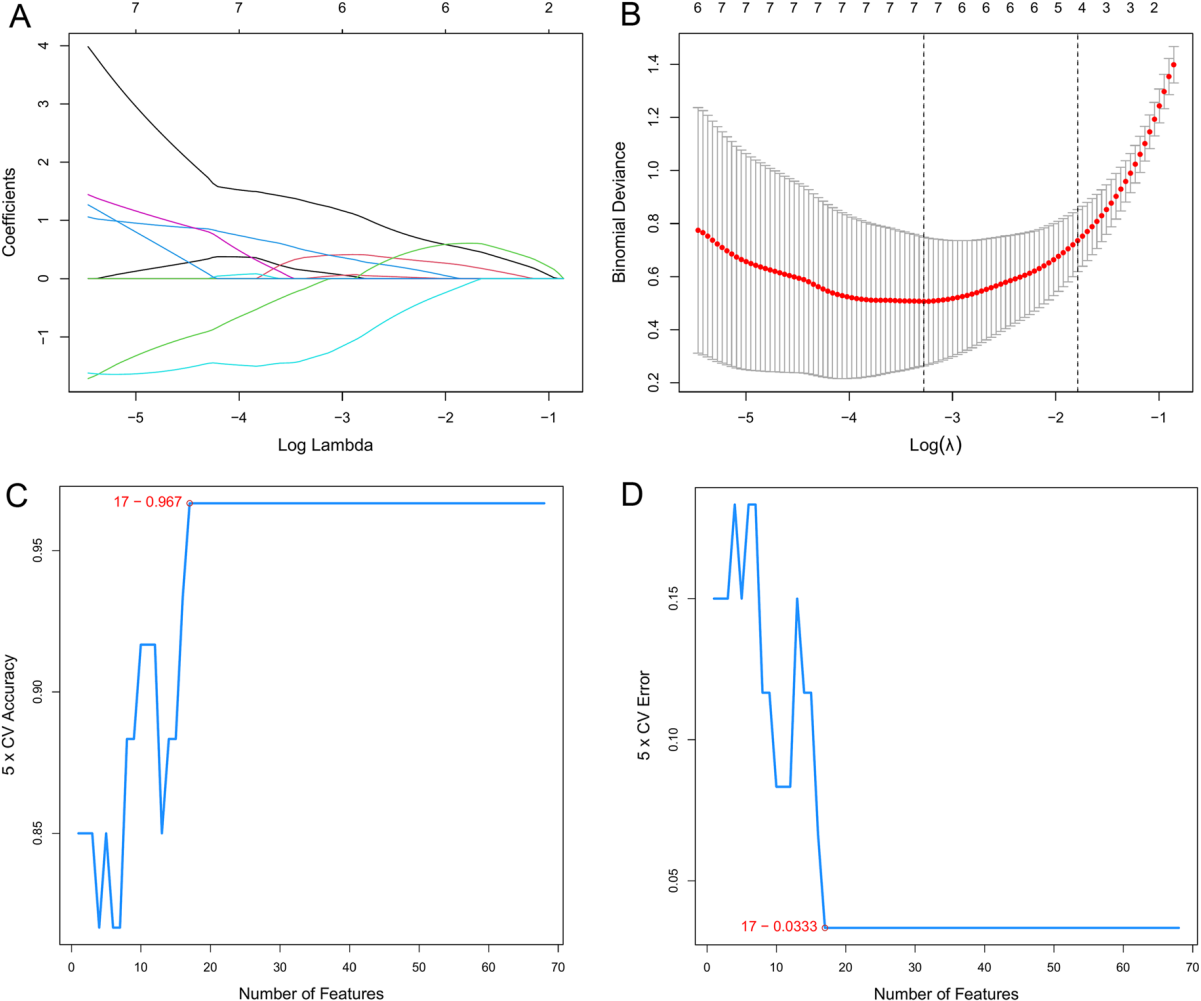
Selection of diagnostic biomarkers using the machine learning methods. **A**, **B** LASSO algorithm to screen candidate genes. **C**, **D** SVM-RFE algorithm to screen candidate genes. The point highlighted indicates the optimal accuracy and the lowest error rate, respectively, and the corresponding genes at this point are the best signature selected by SVM-RFE

**Fig. 5:**
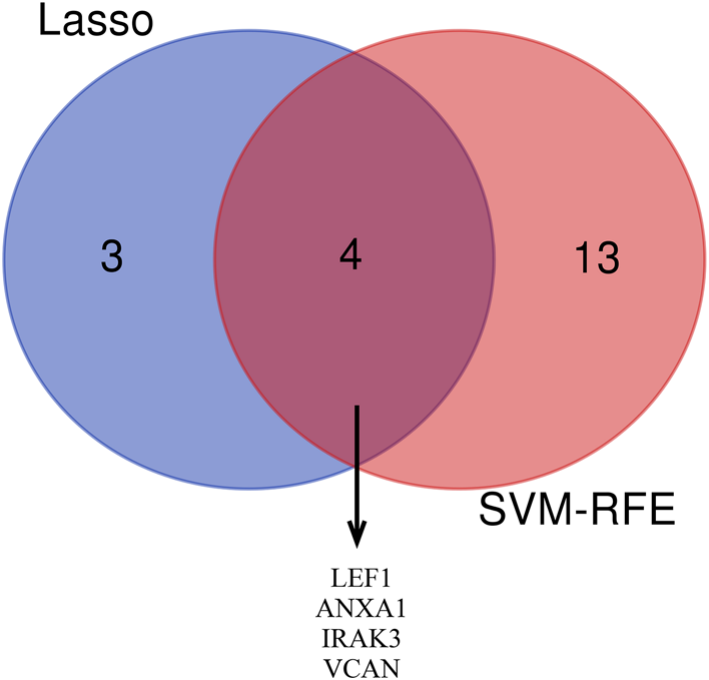
Venn diagram showing the hub genes shared by LASSO and SVM-RFE algorithms

**Fig. 6:**
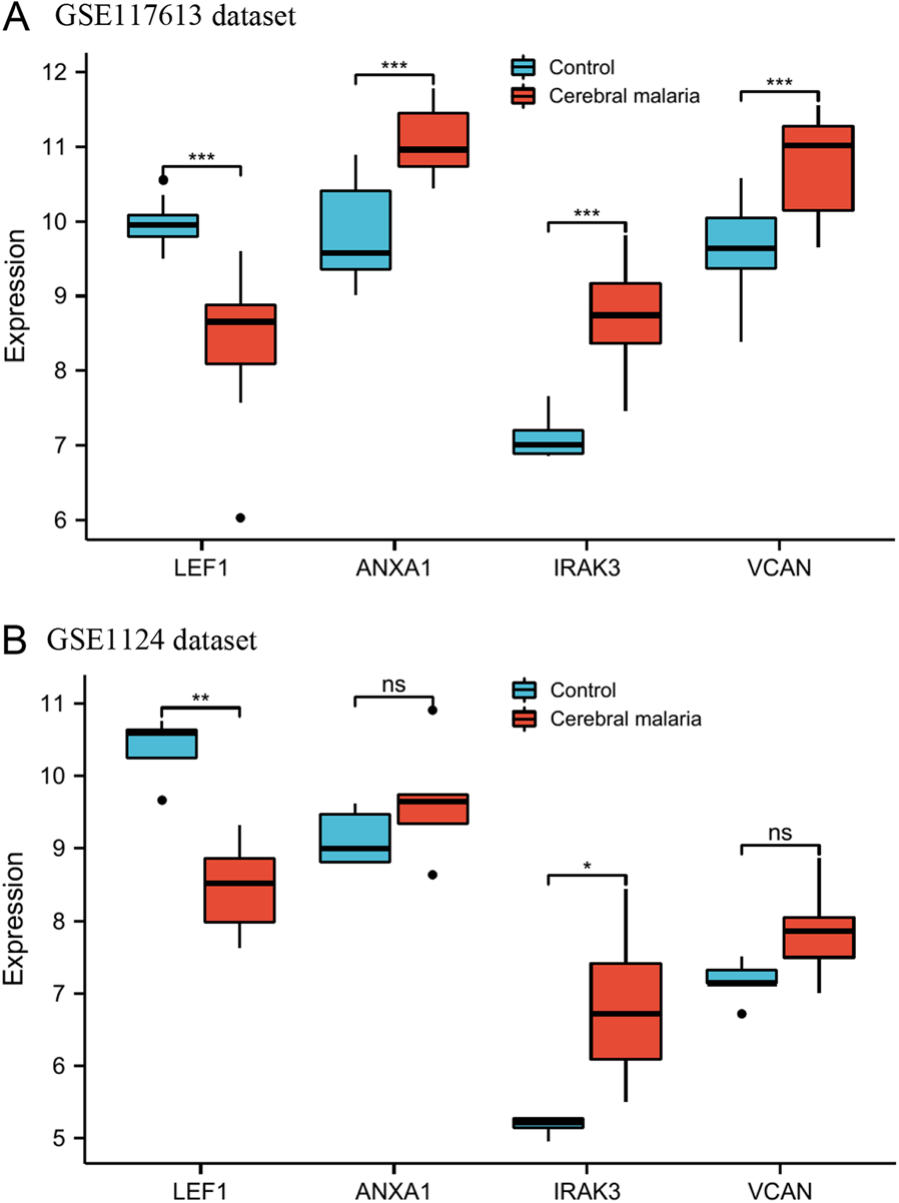
Validation of hub genes in GSE117613 and GSE1124 datasets

To verify the diagnostic value of the genes, 68 candidate genes were subjected to LASSO logistic regression and SVM-RFE algorithms, respectively. LASSO identified 7 genes (Fig. 4A, B), while SVM-RFE screened 17 genes (Fig. 4C, D). The common genes (*LEF1*, *ANXA1*, *IRAK3*, and *VCAN*) obtained by LASSO and SVM-RFE algorithms were identified as hub genes (Fig. 5). Subsequently, we validated the expression levels of four hub genes in GSE117613 and a separate external dataset, GSE1124. Compared to the control group, *IRAK3*, *VCAN*, and *ANXA1* were upregulated, while *LEF1* was downregulated in the CM group in the GSE117613 dataset (Fig. 6). However, only *LEF1* and *IRAK3* had significantly different expression levels in the validation dataset GSE1124. (*p* < 0.05) Thus, *LEF1* and *IRAK3* were identified as hub genes and subjected to subsequent research.

### Gene set enrichment analysis (GSEA)

**Fig. 7:**
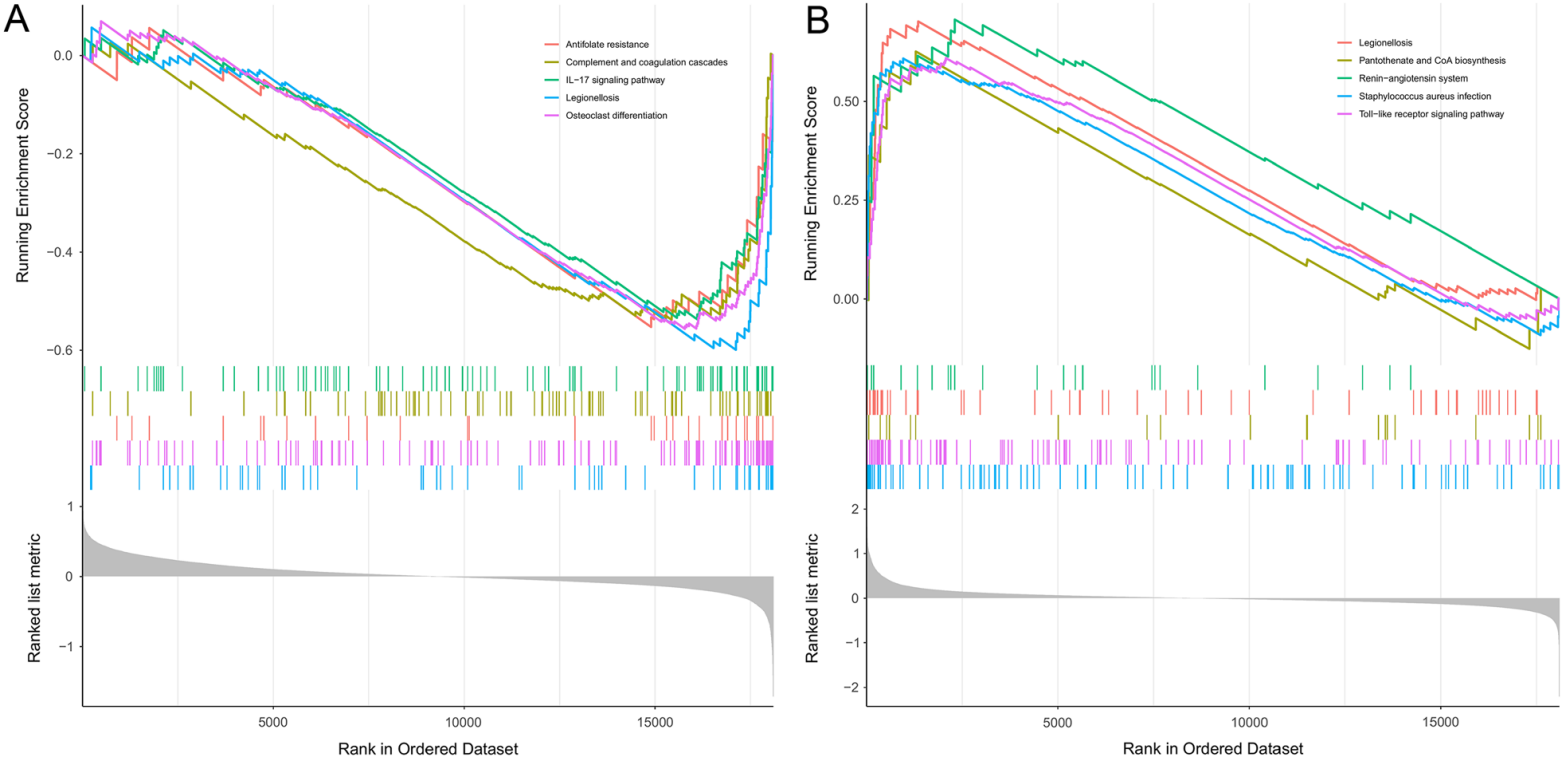
Gene set enrichment analysis of hub genes. **A** The main signaling pathways that are significantly enriched in low-*LEF1* group. **B** The main signaling pathways that are significantly enriched in high-*IRAK3* group

The enriched KEGG pathways are shown in Fig. 7, which may reveal the potential regulatory mechanisms of hub genes among blood in the progression of CM. According to the enrichment results, some immune-related pathways (IL − 17 signaling pathway and Toll-like receptor signaling pathway) were enriched in the low-*LEF1* group and high-*IRAK3* group (Fig. 7A, B).

## Discussion

CM is a life-threatening complication of Plasmodium falciparum [16]. In addition to its high fatality, 10% to 20% of surviving children with CM suffer from severe neurological sequelae [17]. Deciphering the molecular mechanism of malaria is of great significance for early diagnosis and developing new treatment strategies to reduce the burden of CM. Recently, microarray technologies and bioinformatic analyses have become popular methods for exploring disease pathogenesis and identifying biomarkers of disease [18]. The GSE117613 and GSE1124 datasets may provide new insights into the identification of pathophysiology and biomarkers in CM. Some researchers have previously analyzed the GSE117613 and GSE1124 datasets. Nallandhighal, Srinivas et al., who offered the original data for GSE117613, compared the whole-blood transcriptional profile difference between CM and severe malaria anemia and reported their difference in oxidative stress and erythropoietic responses [12]. Boldt, Angelica B W et al. revealed the modifications of gene expression in different stages of P. falciparum infection and identified some potential prognostic markers [19]. Unlike the previous studies mentioned in this report, we utilized WGCNA, a new bioinformatics tool, to investigate the molecular mechanisms underlying CM. By constructing the WGCNA network, we identified the CM-related module and extracted the hub genes. Additionally, the application of machine learning methods contributed to the screening of gene biomarkers with high diagnostic value.

The GO and KEGG enrichment results illustrated the regulating pathways involved in the DEGs. The BP enrichment results revealed that neutrophils and their related pathways played important roles in CM. The results remained the same as those of previous studies. Feintuch et al. reported higher levels of activated neutrophils in malarial retinopathy-positive CM pediatric patients [20]. Some studies hypothesized that neutrophils stimulated by the large numbers of sequestered parasites in the retinal and cerebral microvasculature in CM could degranulate, releasing MMP8 and leading to vascular endothelial barrier damage and vascular leakage [21]. Moreover, these vascular dysfunctions are common features of CM [22]. In terms of CC, DEGs were involved in the form of vesicle lumen. Previous research has shown that iRBCs secrete significantly more extracellular vesicles than uninfected cells [22]. Vesicles contain many poisonous factors, resulting in vascular dysfunction and the severity of disease in CM [23]. For KEGG, the NOD-like signaling pathway is one of the significantly enriched pathways. Some studies [24, 25] reported that hemozoin, a product of hemoglobin (Hb) catabolism, triggered a high level of IL-1β production and recruited neutrophils by activating the NOD-like receptors containing pyrin domain 3 (*NLRP3*) inflammasome complex in malaria infection. High IL-1β was highly associated with disease severity and death during malaria [26, 27]. Previous studies found that the *NLRP3*–IL1β axis was involved in acute cerebrovascular dysfunction and progressive neuroinflammation in various brain pathologies [28, 29]. Therefore, inhibition of the *NLRP3*–IL1β axis may weaken neurological sequelae and improve the efficacy of antimalarial drug treatment of CM [30].

This study also identified some biomarkers for CM by LASSO and SVM-RFE. However, the possible effects of these hub genes in CM have not been reported in previous studies. *LEF1* (Lymphoid Enhancer Binding Factor 1) is a protein-coding gene involved in the Wnt signaling pathway [31]. Several studies [32] have reported that activation of the Wnt signaling pathway contributes to maintaining the integrity of the blood–brain barrier (BBB) in various cerebral diseases. Jin, Zhao et al. found that activation of the Wnt signaling pathway may inhibit MMP-9 activation and upregulate *LEF1* expression, which alleviated BBB breakdown and reduced brain edema in cerebral ischemia–reperfusion [33]. Therefore, we suggest that the downregulation of *LEF1* may imply the absence of the Wnt signaling pathway in CM and poor prognosis. In addition, according to the enriched GSEA terms, the IL-17 signaling pathway was highly enriched in the low-*LEF1* group. It's reported that excess expressions of immune-related pathways were associated with disease development or poor prognosis in CM [34]. Huppert, Jula et al. disclosed that IL-17 is involved in the disruption of the BBB [35], which is frequently fatal and related to long-term neurological sequelae [36]. Therefore, the downregulation of *LEF1* may suggest the disruption of the BBB and the poor prognosis of CM.

*IRAK3* is a member of the interleukin-1 receptor-associated kinase protein family and functions as a negative regulator of Toll-like receptor (TLR) signaling [37]. Dickinson-Copeland, Carmen M et al. reported that the activation of TLR-mediated heme-induced apoptosis, leading to the depletion of endothelial progenitor cells, which contributed to vascular dysfunction and BBB damage [38]. Additionally, *IRAK3* is an important inflammatory down-regulator that reduces the transcription of NF-κB-induced cytokines [39]. NF-κB activation contributes to the cause of apoptosis in endothelial cells in CM, leading to BBB dysfunction [40]. Therefore, we suggest that the high expression of *IRAK3* contributes to maintaining the integrity of the BBB by inhibiting the Toll-like receptor and NF-κB pathways, thereby improving the prognosis of CM.

In this study, we identified *LEF1* and *IRAK3* as important biomarkers in CM. Through various bioinformatic analyses, we validated their diagnostic value in CM and found that they were highly associated with the integrity of the BBB. Therefore, we suggest that *LEF1* and *IRAK3* may act as key targets to improve prognosis, contributing to the diagnosis and treatment of CM.

We acknowledge that there are some limitations in our study. First, the datasets included in our study did not have enough samples. In addition, in vitro and in vivo experiments are required to further validate the value of hub genes in CM.

## Supplementary Information

**Additional file 1: Figure S1.** Sample clustering dendrogram.

**Additional file 2: Figure S2.** The network topology analysis for various soft-thresholding powers.

## Data Availability

The datasets analyzed in our study are available in the Gene Expression Omnibus repository (https://www.ncbi.nlm.nih.gov/geo).

## References

[1] Tu Z, Gormley J, Sheth V (2021). Cerebral malaria: insight into pathology from optical coherence tomography. Sci Rep.

[2] Mturi N, Musumba CO, Wamola BM, Ogutu BR, Newton CR (2003). Cerebral malaria: optimising management. CNS Drugs.

[3] Storm J, Craig AG (2014). Pathogenesis of cerebral malaria–inflammation and cytoadherence. Front Cell Infect Microbiol.

[4] Polimeni M, Prato M (2014). Host matrix metalloproteinases in cerebral malaria: new kids on the block against blood-brain barrier integrity?. Fluids Barriers CNS.

[5] Vanka R, Nakka VP, Kumar SP, Baruah UK, Babu PP (2020). Molecular targets in cerebral malaria for developing novel therapeutic strategies. Brain Res Bull.

[6] Song X, Wei W, Cheng W (2022). Cerebral malaria induced by plasmodium falciparum: clinical features, pathogenesis, diagnosis, and treatment. Front Cell Infect Microbiol.

[7] Patel H, Dunican C, Cunnington AJ (2020). Predictors of outcome in childhood plasmodium falciparum malaria. Virulence.

[8] Lucchi NW, Jain V, Wilson NO, Singh N, Udhayakumar V, Stiles JK (2011). Potential serological biomarkers of cerebral malaria. Dis Markers.

[9] Sahu PK (2015). Pathogenesis of CM: new diagnostic tools, biomarkers, and therapeutic approaches. Front Cell Infect Microbiol.

[10] Noedl H (2008). Evidence of artemisinin-resistant malaria in western Cambodia. N Engl J Med.

[11] Fairhurst RM, Dondorp AM (2016). Artemisinin-resistant plasmodium falciparum malaria. Microbiol Spectr.

[12] Nallandhighal S, Park GS, Ho YY, Opoka RO, John CC, Tran TM (2019). Whole-blood transcriptional signatures composed of erythropoietic and NRF2-regulated genes differ between CM and severe malarial anemia. J Infect Dis.

[13] Langfelder P, Horvath S (2008). WGCNA: an R package for weighted correlation network analysis. BMC Bioinform.

[14] Friedman J, Hastie T, Tibshirani R (2010). Regularization paths for generalized linear models via coordinate descent. J Stat Softw.

[15] Guyon I, Weston J, Barnhill S (2002). Gene selection for cancer classification using support vector machines. Mach Learn.

[16] O'Sullivan JM, Preston RJ, O'Regan N, O’Donnell JS (2016). Emerging roles for hemostatic dysfunction in malaria pathogenesis. Blood.

[17] Birbeck GL, Molyneux ME, Kaplan PW, Seydel KB, Chimalizeni YF, Kawaza K, Taylor TE (2010). Blantyre malaria project epilepsy study (BMPES) of neurological outcomes in retinopathy-positive paediatric CM survivors: a prospective cohort study. Lancet Neurol.

[18] Tang YL, Fang LJ, Zhong LY, Jiang J, Dong XY, Feng Z (2020). Hub genes and key pathways of traumatic brain injury: bioinformatics analysis and in vivo validation. Neural Regen Res.

[19] Boldt ABW, van Tong H, Grobusch MP (2019). The blood transcriptome of childhood malaria. EBioMedicine.

[20] Feintuch CM, Saidi A, Seydel K, Chen G, Goldman-Yassen A, Mita-Mendoza NK, Kim RS, Frenette PS, Taylor T, Daily JP (2016). Activated neutrophils are associated with pediatric CM vasculopathy in malawian children. MBio.

[21] Georgiadou A, Naidu P, Walsh S, Kamiza S, Barrera V, Harding SP, Moxon CA, Cunnington AJ (2021). Localised release of matrix metallopeptidase 8 in fatal CM. Clin Transl Immunol.

[22] Mantel PY, Hjelmqvist D, Walch M, Kharoubi-Hess S, Nilsson S, Ravel D, Ribeiro M, Grüring C, Ma S, Padmanabhan P, Trachtenberg A, Ankarklev J, Brancucci NM, Huttenhower C, Duraisingh MT, Ghiran I, Kuo WP, Filgueira L, Martinelli R, Marti M (2016). Infected erythrocyte-derived extracellular vesicles alter vascular function via regulatory Ago2-miRNA complexes in malaria. Nat Commun.

[23] El-Assaad F, Wheway J, Hunt NH, Grau GE, Combes V (2014). Production, fate and pathogenicity of plasma microparticles in murine CM. PLoS Pathog.

[24] Olivier M, Van Den Ham K, Shio MT, Kassa FA, Fougeray S (2014). Malarial pigment hemozoin and the innate inflammatory response. Front Immunol.

[25] Griffith JW, Sun T, McIntosh MT, Bucala R (2009). Pure Hemozoin is inflammatory in vivo and activates the NALP3 inflammasome via release of uric acid. J immunol.

[26] Schofield L, Grau GE (2005). Immunological processes in malaria pathogenesis. Nat Rev Immunol.

[27] Kalantari P, DeOliveira RB, Chan J, Corbett Y, Rathinam V, Stutz A, Latz E, Gazzinelli RT, Golenbock DT, Fitzgerald KA (2014). Dual engagement of the NLRP3 and AIM2 inflammasomes by plasmodium-derived hemozoin and DNA during malaria. Cell Rep.

[28] Murray KN, Parry-Jones AR, Allan SM (2015). Interleukin-1 and acute brain injury. Front Cell Neurosci.

[29] Song L, Pei L, Yao S, Wu Y, Shang Y (2017). NLRP3 Inflammasome in neurological diseases, from functions to therapies. Front Cell Neurosci.

[30] Strangward P, Haley MJ, Albornoz MG, Barrington J, Shaw T, Dookie R, Zeef L, Baker SM, Winter E, Tzeng TC, Golenbock DT, Cruickshank SM, Allan SM, Craig A, Liew FY, Brough D, Couper KN (2018). Targeting the IL33-NLRP3 axis improves therapy for experimental CM. Proc Natl Acad Sci USA.

[31] Eastman Q, Grosschedl R (1999). Regulation of LEF-1/TCF transcription factors by Wnt and other signals. Curr Opin Cell Biol.

[32] Polakis P (2008). Formation of the blood-brain barrier: Wnt signaling seals the deal. J Cell Biol.

[33] Jin Z, Ke J, Guo P, Wang Y, Wu H (2019). Quercetin improves blood-brain barrier dysfunction in rats with cerebral ischemia reperfusion via Wnt signaling pathway. Am J Transl Res.

[34] Brant F, Miranda AS, Esper L (2016). Suppressor of cytokine signaling 2 modulates the immune response profile and development of experimental CM. Brain Behav Immun.

[35] Huppert J, Closhen D, Croxford A (2010). Cellular mechanisms of IL-17-induced blood-brain barrier disruption. FASEB J.

[36] Adams Y, Jensen AR (2022). CM—modelling interactions at the blood-brain barrier in vitro. Dis Model Mech.

[37] Kobayashi K, Hernandez LD, Galán JE, Janeway CA, Medzhitov R, Flavell RA (2002). IRAK-M is a negative regulator of Toll-like receptor signaling. Cell.

[38] Dickinson-Copeland CM, Wilson NO, Liu M (2015). Heme-Mediated induction of CXCL10 and depletion of CD34+ progenitor cells Is toll-like receptor 4 dependent. PLoS ONE.

[39] Freihat LA, Wheeler JI, Wong A, Turek I, Manallack DT, Irving HR (2019). IRAK3 modulates downstream innate immune signalling through its guanylate cyclase activity. Sci Rep.

[40] Punsawad C, Maneerat Y, Chaisri U, Nantavisai K, Viriyavejakul P (2013). Nuclear factor kappa B modulates apoptosis in the brain endothelial cells and intravascular leukocytes of fatal CM. Malar J.

