# Supplementary figures and images for: Identifying effective diagnostic biomarkers for childhood cerebral malaria in Africa integrating coexpression analysis with machine learning algorithm
Source: Eur J Med Res. 2023 Feb 13;28:76. doi: 10.1186/s40001-022-00980-w (PMC9926768; doi:10.1186/s40001-022-00980-w)

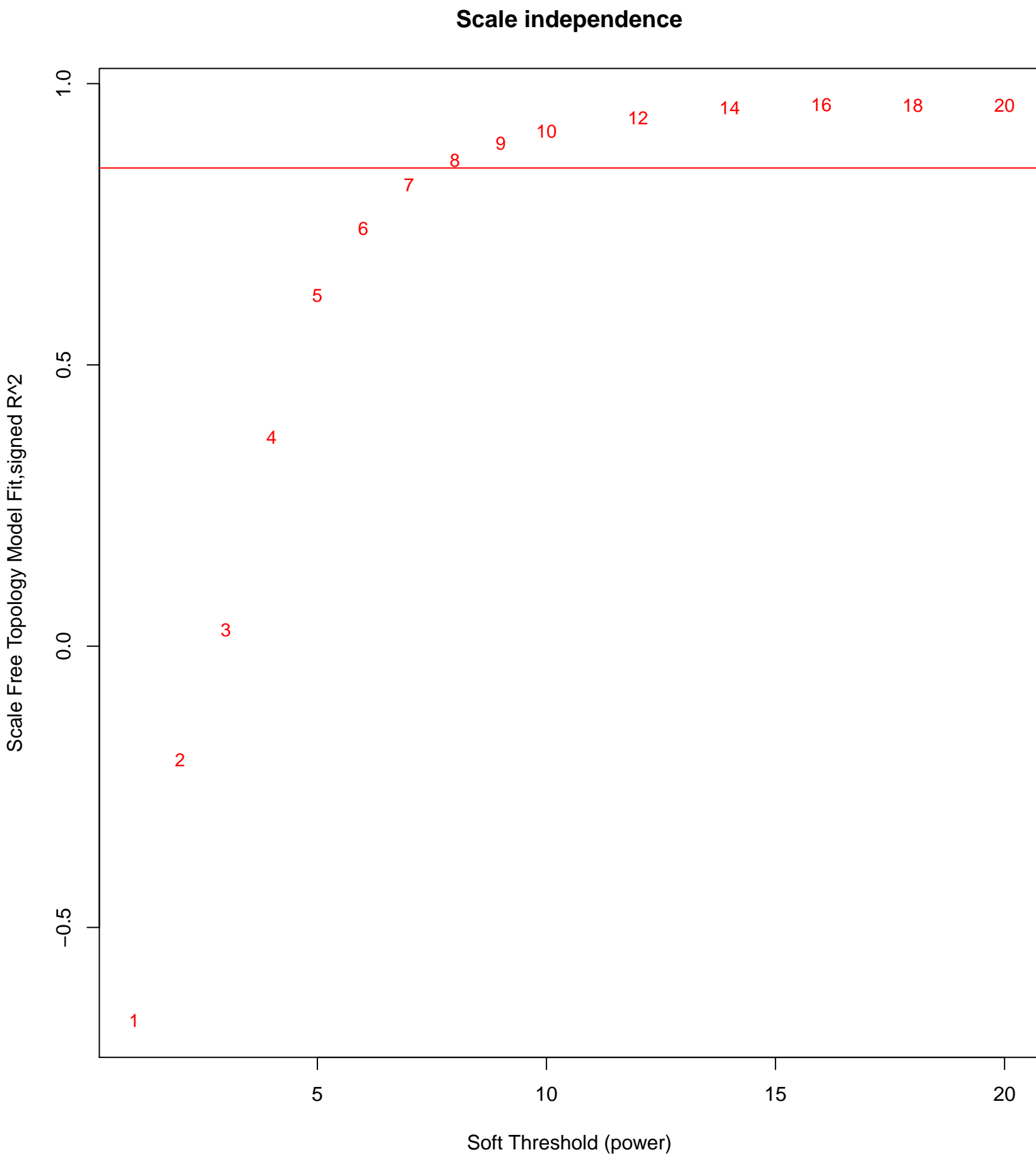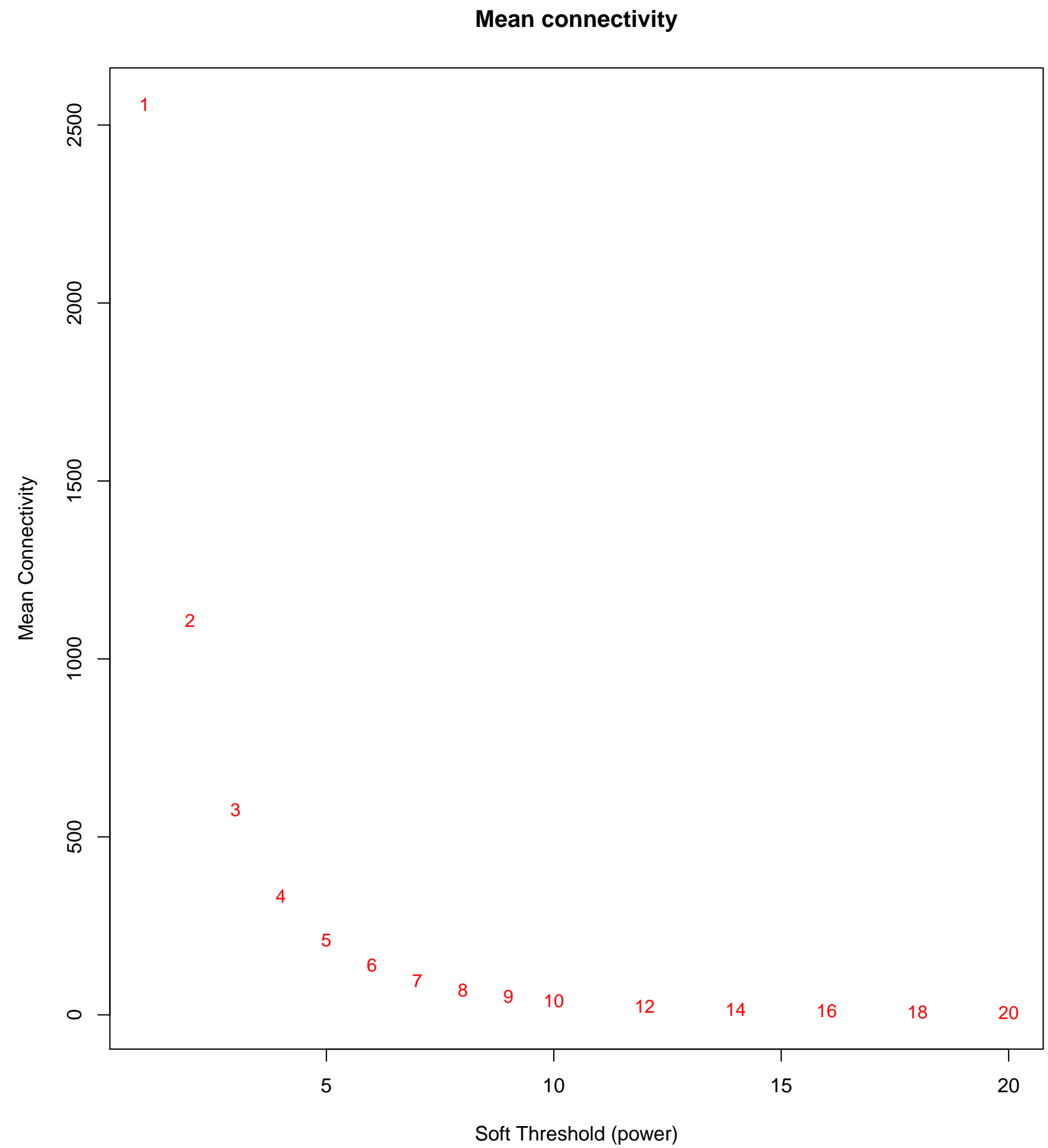

Supplement: Supplementary file 1 — Additional file 1: Figure S1. Sample clustering dendrogram. [file 40001_2022_980_MOESM1_ESM.pdf]

Sample clustering to detect outliers

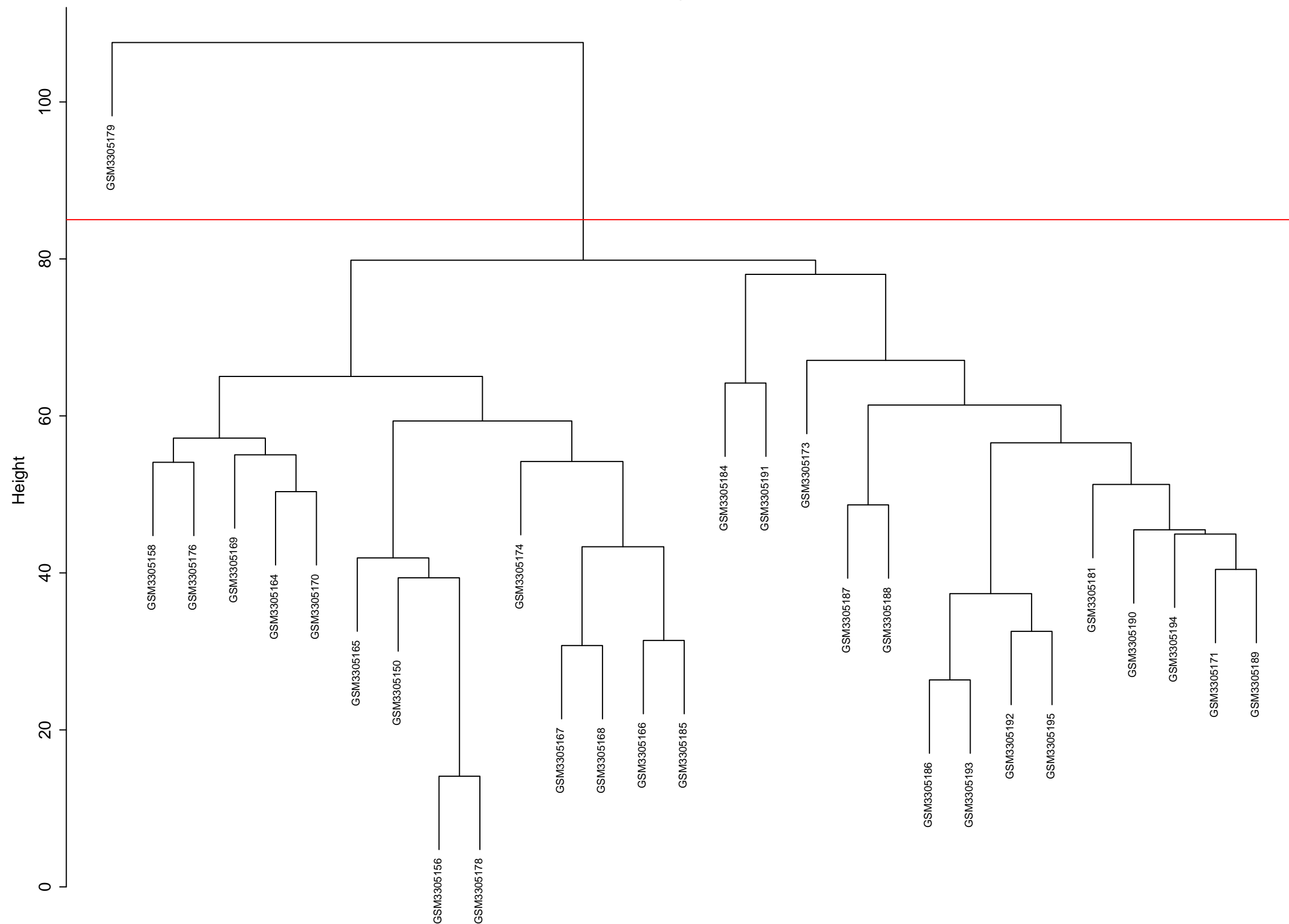

Supplement: Supplementary file 2 — Additional file 2: Figure S2. The network topology analysis for various soft-thresholding powers. [file 40001_2022_980_MOESM2_ESM.pdf]
